# The Problem of the Town-Bred Child

**Published:** 1918-03-30

**Authors:** 


### PHYSICAL CULTURE IN EDUCATION.

### II.—The Problem of the Town-bred Child.

The progressive urbanisation of the population in this and every "civilised" country has led to the propagation and perpetuation of many physical defects, most marked, of course, among the poorer classes, as well as a large increase in infantile mortality, directly to be attributed to faulty conditions of housing and sanitation.

With the problems of the infant death-rate we have here no concern, since we aim rather at considering the various remedial measures adopted to combat the physical degeneracy that is at present the inevitable concomitant of urban life. "City life," says Dr. Tait Mackenzie, "means biologic death. People multiply like bacteria in a culture medium until they poison themselves with their own waste. Their marriage-rate falls, the birth-rate falls, and the death-rate rises, until the race would die out were it not kept up by a constant influx from the country and from foreign lands."

### The Significance of the Housing Problem.

A consideration of the housing conditions in this country as revealed by the Census of 1911 shows a condition of affairs that is truly appalling. Of the total population 2,580,814, or 7.1 per cent., were living in one-or two-roomed dwellings, and 4,429,119, or 12.3 per cent., in three-roomed dwellings. Satisfactory family life, even for a healthy family, is difficult to maintain under the above conditions, and in the case of growing children, unless some outlet be found for the expanding energies of youth, the nation has to bear in after-years the burden of a vast amount of avoidable sickness and chronic invalidism. The systematic examination of school children has done much towards the eradication of remedial defects. Enlarged tonsils and adenoids, that most fruitful source of impaired mental and physical development, are now detected early; errors of refraction are corrected; tuberculous joints and tabes mesenterica each demand and receive adequate specialist treatment. But, as Samuel Butler long ago pointed out, health is a positive condition of well-being, not merely a state in which one is not ill; and though a child may not be suffering from any well-marked disorder, it may yet fall far short of an ideal standard of growth and vitality.

The only remedy, short of decentralisation, which is going to make the vitiated slum dweller approximate in physique to the Canadian and Australian soldiers who at present fill the streets seems to be the provision of some organised system of municipal playing grounds, where free play on scientifically constructed apparatus will replace the haphazard diversions of the alley and street corner.

Let us consider how America has approached the problem.

### The American Playground Association.

A century ago only 4 per cent. of the population in America lived in towns, whilst in 1900 the urban population was over 30 per cent. So rapid a rate of increase means necessarily, or almost so, the hasty construction of ill-planned dwellings, and in point of fact many American cities are notoriously bad in this respect.

Realising the evil issues of slum life, the American Playground Association was established in 1906 to unite the various local bodies into one great concerted campaign, to interest every American city in playgrounds, to collect literature upon the subject of children's games, and found courses in normal schools for the training of play supervisors.

From this date a regular plan of campaign has been instituted; facts and figures are collected to impress the city fathers with the desirability from a moral as well as a physical standpoint of purchasing vacant plots of land in crowded areas as playgrounds and recreation centres for the young people of both sexes. For example, a record of twenty-one months in New York shows 46 children between one and sixteen killed by automobiles, 140 injured; 20 killed and 17 injured by trolley cars; 104 killed and 23 injured by wagons. The provision of playgrounds would have averted the greater number of these disasters.

Moreover, when the energies of youth are allowed free play amid the temptations of the streets, pilfering, acts of violence by organised gangs, and sexual precocity are the inevitable results. It is stated that the opening of a small park, such as those upon the South Side of Chicago, coincides with a 28 per cent. decrease of juvenile delinquency within the radius of half a mile, and with a 17 per cent. decrease over a larger area.

### Playgrounds to Suit all Conditions.

Following the American classification, playgrounds are divisible into three groups, according to size. The smallest are those obtained by the utilisation of any vacant plot of land, and are equipped at small cost with simple apparatus suitable for young children. Such an outfit includes two swings ten feet in height, for larger children; two six-foot swings with safety cradles for younger children to sleep or swing in; two or three see-saws, a sand box filled with fine white sand for the children to dig in, an awning to provide shelter from rain and sun.

The average cost is about eighty or ninety dollars (£16 to £18). A doubt may be expressed as to the advisability of the sand pile, for experience in London parks has shown it to be a fruitful breeding ground for vermin of all sorts. A wading pool is added wherever means and circumstances allow. An endeavour is being made to establish these playing grounds in every two or three blocks, so that the children do not need to go more than a few hundred yards from their homes.

Playgrounds of the second type are larger in area (2 to 10 acres), and contain a more elaborate provision for outdoor and indoor games.

In the centre is a framework for the fixed gymnastic apparatus, ladders, rings, ropes, parallel bars, and vaulting horses of various types. A wading pool is provided for the little ones, a swimming bath for those who are older. Where space permits there is a running track around the field. Playing grounds such as these are necessarily for older boys and girls, ranging from twelve to eighteen, but a space is set apart for the smaller children.

### Standard Tests of Efficiency.

The third type has examples in Central Park, New York, Boston Common, and Fairmount Park, Philadelphia. Here are innumerable tennis courts, room for

☛ The First Article appeared Jan 12, p. 312.

**PHYSICAL CULTURE IN EDUCATION—(*continued*).**

two or more games of baseball, a swimming pool—that in Chicago can be entered only by a room containing fifteen shower baths, where the bathers are inspected before entering—and a quarter-mile running track. In addition a large pavilion is generally available for lectures, dances, and indoor contests.

These parks become the social centre of the district in which they are situated. A special series of tests has been devised by the American Playground Association, which every well-built boy should be able to do. At twelve he is expected to "pull up" four times, clear five feet nine inches in a standing broad jump, and run sixty yards in eight and three-fifth seconds. A year later he must "pull up" six times, run 100 yards in fourteen seconds, and clear six feet six inches. At fifteen more difficult tests are imposed. The passing of each grade is rewarded by a medal, engraved with an appropriate Greek design.

